# Supplementary material for: Comparison of the New Neo-Glasgow Prognostic Score Based on the Albumin-Bilirubin Grade with Currently Used Nutritional Indices for Prognostic Prediction following Surgical Resection of Hepatocellular Carcinoma: A Multicenter Retrospective Study in Japan
Source: Cancers (Basel). 2022 Apr 22;14(9):2091. doi: 10.3390/cancers14092091 (PMC9105166; doi:10.3390/cancers14092091)
Supplement: Supplementary file 1 [file cancers-14-02091-s001.zip › cancers-1688796-supplementary.pdf]

Supplementary Materials

Comparison of the New Neo-Glasgow Prognostic Score Based on the Albumin-Bilirubin Grade with Currently Used Nutritional Indices for Prognostic Prediction Following Surgical Resection of Hepatocellular Carcinoma: A Multicenter Retrospective Study in Japan

Masaki Kaibori, Atsushi Hiraoka, Hiroya Iida, Koji Komeda, Fumitoshi Hirokawa, Masaki Ueno, Hisashi Kosaka, Kosuke Matsui and Mitsugu Sekimoto

Table S1. Tumor-Node-Metastasis staging by the Liver Cancer Group Study Group of Japan, 6th edition.

T factor * for TNM stage of LCSGJ, 6th edition
T1: fulfilling three factors
T2: fulfilling two factors
T3: fulfilling one factor
T4: fulfilling no factor
TNM stages in LCSGJ, 6th edition
Stage I: T1N0M0
Stage II: T2N0M0
Stage III: T3N0M0
Stage IVa: T4N0M0 or any TN1M0
Stage IVb: Any TN0-N1M1

LCSGJ: Liver Cancer Study Group of Japan, TNM stage: tumor node metastasis stage, * The three factors are single lesion, lesion measuring <2 cm, and no vascular involvement.

© 2022 by the authors. Licensee MDPI, Basel, Switzerland. This article is an open access article distributed under the terms and conditions of the Creative Commons Attribution (CC BY) license (http://creativecommons.org/licenses/by/4.0/).
